# NMR metabolomics of fibroblasts with inherited mitochondrial Complex I mutation reveals treatment-reversible lipid and amino acid metabolism alterations

**DOI:** 10.1007/s11306-018-1345-9

**Published:** 2018-03-22

**Authors:** Daniel Morvan, Aicha Demidem

**Affiliations:** 1 UCA University, boulevard François Mitterrand, 63001 Clermont-Ferrand, France; 2 Comprehensive Cancer Centre Jean Perrin, rue Montalembert, 63011 Clermont-Ferrand, France; 3 Faculty of Pharmacy, UMR1019 INRA/UCA, ECREIN, place Henri Dunant, 63001 Clermont-Ferrand, France; 4 Department of Biophysics, Faculty of Medicine, Place Henri Dunant, 63001 Clermont-Ferrand, France

**Keywords:** Mitochondrial Complex I deficiency, Leber’s hereditary optic neuropathy, NMR-based metabolomics, Lipid and amino acid metabolism, Idebenone, Resveratrol

## Abstract

**Introduction:**

Elucidating molecular alterations due to mitochondrial Complex I (CI) mutations may help to understand CI deficiency (CID), not only in mitochondriopathies but also as it is caused by drugs or associated to many diseases.

**Objectives:**

CID metabolic expression was investigated in Leber’s hereditary optic neuropathy (LHON) caused by an inherited mutation of CI.

**Methods:**

NMR-based metabolomics analysis was performed in intact skin fibroblasts from LHON patients. It used several datasets: one-dimensional ^1^H-NMR spectra, two-dimensional ^1^H-NMR spectra and quantified metabolites. Spectra were analysed using orthogonal partial least squares-discriminant analysis (OPLS-DA), and quantified metabolites using univariate statistics. The response to idebenone (IDE) and resveratrol (RSV), two agents improving CI activity and mitochondrial functions was evaluated.

**Results:**

LHON fibroblasts had decreased CI activity (− 43%, p < 0.01). Metabolomics revealed prominent alterations in LHON including the increase of fatty acids (FA), polyunsaturated FA and phosphatidylcholine with a variable importance in the prediction (VIP) > 1 in OPLS-DA, p < 0.01 in univariate statistics, and the decrease of amino acids (AA), predominantly glycine, glutamate, glutamine (VIP > 1) and alanine (VIP > 1, p < 0.05). In LHON, treatment with IDE and RSV increased CI activity (+ 40 and + 44%, p < 0.05). IDE decreased FA, polyunsaturated FA and phosphatidylcholine (p < 0.05), but did not modified AA levels. RSV decreased polyunsaturated FA, and increased several AA (VIP > 1 and/or p < 0.05).

**Conclusion:**

LHON fibroblasts display lipid and amino acid metabolism alterations that are reversed by mitochondria-targeted treatments, and can be related to adaptive changes. Findings bring insights into molecular changes induced by CI mutation and, beyond, CID of other origins.

**Electronic supplementary material:**

The online version of this article (10.1007/s11306-018-1345-9) contains supplementary material, which is available to authorized users.

## Introduction

Mitochondrial Complex I (NADH: ubiquinone oxidoreductase, CI) is a multimeric enzyme of the mitochondrial electron transport chain (ETC). CI subunit mutations, carried by either the nuclear or the mitochondrial genome, impair CI activity and are responsible for the most frequent mitochondrial diseases. Elucidating biochemical alterations in these diseases may help to understand CI deficiency (CID), not only in mitochondrial diseases, but also as it is caused by drugs targeting CI, or is a pathogenic component of diseases including neurodegeneration and cancer (Mimaki et al. 2012). To this aim, we investigated using metabolomics Leber’s hereditary optic neuropathy (LHON), a mitochondrial disease caused by an inherited mutation of a mitochondrial DNA-encoded subunit of CI. LHON is a systemic disease although its main clinical expression is blindness associated to the loss of retinal ganglion cells (RGC) through a mechanism incompletely elucidated.

Metabolic abnormalities reported in LHON are few and include decreased CI activity, impairment of CI-driven respiration, increased ROS production, and decreased expression of excitatory amino acid transporter-1 (EAAT1) (Beretta et al. 2004). However, cells with CID have been shown to develop mechanisms of adaptation, including increased mitogenesis that may yield incomplete penetrance of the disease in LHON and AMP-activated protein kinase (AMPK) activation that improves cellular bioenergetics (Distelmaier et al. 2015).

Metabolomics can be expected to improve knowledge about molecular changes associated to CID. However, metabolomics studies of CID are few. A NMR-based metabolomics study on pharmacological inhibition of CI showed the decrease of amino acids (AA) and perturbations of phospholipid metabolism (Baykal et al. 2008). Another NMR study of myotubes exposed to rotenone showed changes in AA levels and increase in lactate (Lac) production (Xu et al. 2011). Using mass spectrometry (MS)-based metabolomics, it was shown that CI mutation in worms caused the decrease of most AA and succinate (Morgan et al. 2015). CI mutation in mice caused the increase of hydroxyacylcarnitines, in favor of decreased betaoxidation (Leong et al. 2012). A MS investigation in LHON fibroblasts reported the decrease of proteinogenic AA and changes in phospholipids (Chao de la Barca et al. 2016). A MS study of cells exposed to pharmacological inhibition of CI showed the decrease of glutamate (Glu), glutamine (Gln) and TCA cycle derivatives, and the increase of Lac (Janzer et al. 2014).

We here report an untargeted NMR-based metabolomics analysis of intact skin fibroblasts from LHON patients. LHON metabolomics is challenged by the low availability of samples and biological variability associated to the disease. We thus used orthogonally-filtered partial least squares discriminant analysis (OPLS-DA) in an attempt to reveal metabolic changes specifically related to LHON. In this study, OPLS-DA was applied to high resolution one-dimensional (1D) ^1^H-NMR spectra and two-dimensional (2D) ^1^H-NMR spectra whenever available. In addition, a set of metabolites was quantified from 1D ^1^H-NMR spectra.

Treatment of LHON remains a challenge. At present mitochondria-targeted agents remain the most beneficial. One of the most employed, idebenone (IDE), favors bypassing of CI directly to Complex III of the ETC (Angebault et al. 2011). Another beneficial agent, resveratrol (RSV), improves antioxidant defences in CI mutations (Mathieu et al. 2016) and protects against rotenone-induced apoptosis and neurodegeneration (Zhang et al. 2015). We thus sought for evaluating metabolic changes induced in LHON fibroblasts by IDE and RSV.

In this article, it is shown that LHON fibroblasts exhibit two prominent alterations, the increase of lipids and the decrease of AA, which are reversed by treatments. These data shed light on metabolic and molecular alterations in CID.

## Materials and methods

### Patients

Patient demographic and genetic data are given in Table S1. The study was approved by the Ethics Committee of the University Hospital of Angers (No. 2011/39). All patients gave informed consent. A skin biopsy was obtained from 5 affected LHON patients and 4 healthy subjects. Healthy subjects were males, aged between 31 and 59, without any personal or familial history of neurological or genetic disease.

### Cell culture and treatment

Fibroblasts were cultured according to a published protocol (Loiseau et al. 2007). Ethanol, used as the vehicle, idebenone (IDE, Santhera Pharmaceutical, Liestal, Switzerland) at 10 µM, and resveratrol (RSV, Sigma Chemicals, St. Louis, MO) at 50 µM were added 24 h before cell collection. Doses of IDE and RSV were selected from literature data (Angebault et al. 2011; Mathieu et al. 2016).

For NMR spectroscopy analysis, 5–10 × 10^6^ cells were washed twice in 500 µL D_2_O, then frozen in liquid nitrogen and kept at − 20 °C until analysis. Primary cell cultures were performed twice with vehicle-exposed NL fibroblasts (n = 8 samples from 4 healthy subjects) and LHON patient fibroblasts (n = 10 from 5 patients). Other LHON fibroblast cultures were treated with IDE (L-IDE, n = 8) or RSV (L-RSV, n = 9) and compared to NL fibroblast cultures treated with IDE (N-IDE, n = 7) or RSV (N-RSV, n = 6).

### Complex I activity measurement

Complex I (CI, NADPH ubiquinone reductase, EC 1.6.5.3) activity was measured according to a procedure described elsewhere (Loiseau et al. 2007). Citrate synthase (CS, EC 2.3.3.1) activity was assayed by a standard procedure, and used to correct CI activity for mitochondrial mass in the CI-to-CS activity ratio.

### NMR spectroscopy

NMR spectroscopy was performed on a small-bore 500-MHz Bruker Avance DRX spectrometer equipped with a high resolution magic angle spinning (HRMAS) probe enabling to analyze intact cells. Freshly unfrozen cell pellets were set into 4 mm-diameter, 50-µL zirconium oxide rotor tubes. The rotors were spun at 4 kHz and maintained at 4 °C, using the BCU-05 temperature unit. One-dimensional ^1^H-NMR spectra were obtained using a nuclear Overhauser enhancement spectroscopy sequence with low-power water-signal presaturation (NOESYPR) during both the 3.8-s relaxation delay and the 100-ms mixing time of the sequence. The spectral width was 12 ppm with 16,384 complex data points and 32 transients.

A subset of intact cell pellets kept at 4 °C underwent a 2D ^1^H-NMR Total Correlation Spectroscopy (TOCSY) sequence with water signal suppression at low power, 6-ppm spectral bandwidth along both axes, 256 × 2048 matrix, 75-ms mixing time, during which was applied the spin-lock pulse train (DIPSI-2), 1-s relaxation delay, and 16 repetitions.

### Data processing

One dimensional NMR spectra were transferred to the MestReNova software (Mestrelab Research, Santiago de Compostela, Spain). A standardized phase correction was applied. The residual water signal was suppressed between 4.5 and 5.3 ppm. Strongly contributing signals of HEPES (between 3.08 and 3.19 ppm, and between 3.85 and 3.92 ppm) and ethanol (1.18 and 3.62 ppm) were removed. Then spectra at full resolution were normalized to the total spectral area, and transferred to the SIMCA 14 software (Umetrics, Umea, Sweden) for data processing.

TOCSY spectra were reconstructed at 256 × 256 then transferred to the Matlab 7 software (Mathworks, Natik, MA). The residual water signal was removed from spectra along both spectral axes. TOCSY spectra were normalized to the total spectral area, then linearized and transferred to the SIMCA 14 software for data processing. OPLS-DA results were reconstructed into 2D matrices along spectral coordinates using Matlab 7.

As a third set of NMR data, metabolites giving rise to signals without superposition (or with easy to handle superposition), or with low intensity but sufficient signal-to-noise ratio in 1D NMR spectra were quantified (n = 21). The attribution of signals was based on literature data. Signals were integrated using MestReNova. Metabolite relative concentrations were calculated by normalizing the metabolite signal to the residual Lys protein signal at 2.99 ppm, by adaptation of a technique developed for 2D ^1^H NMR HRMAS quantification (Bayet-Robert et al. 2010).

### Statistical data analysis

Data was scaled using the unit variance method, then processed by Orthogonal Partial Least Squares Discriminant Analysis (OPLS-DA) using the SIMCA 14 software (Umetrics, Uppsala, Sweden).

First OPLS-DA removes data that is not correlated to classes. Remaining data is fitted to a linear combination of variables. The obtained component or predictive component is tested for significance using a permutation algorithm (Monte-Carlo cross validation algorithm) by the SIMCA 14 software. If it is significant, other components, orthogonal to the first component and between one another, are generated as long as they are statistically significant, based on the built-in cross-validation test of the SIMCA 14 software.

The overall quality of the model is described by R2X, R2Y and Q2 parameters that designate the explained fraction of variance of data (R2X), the explained fraction of variance of classes (R2Y), and the cross-validated fraction of variance of classes (Q2), respectively.

For each analysis, OPLS-DA provided:

(i) A scores plot (t[1] vs. t[0]) displaying individuals projected on the first two components.
(ii) A loadings plot (p[1]), displaying variables projected on the first component.
(iii) A correlation table (p(corr)[1]), giving the correlation coefficient of scores with data for each variable. A plot of un-normalized p[1] loadings against the chemical shift, combined with p(corr)[1] was used to display 1D ^1^H-NMR spectrum-derived OPLS-DA models. Variables that combine high strength p[1] and pcorr[1] contribute the most to the model.
(iv) A variable importance for the projection (VIP) table. VIP > 1 was the criterion for statistical significance of a variable.

Univariate statistical analysis used the unpaired Student’s *t* test. Differences were considered significant at p < 0.05.

## Results

### LHON fibroblasts exhibit increased lipid and decreased amino acid levels

The CI-to-CS activity ratio was decreased in LHON (62 ± 11 vs. 109 ± 7%, LHON vs. NL, p < 0.01) (Fig. S1).

**Fig. 1:**
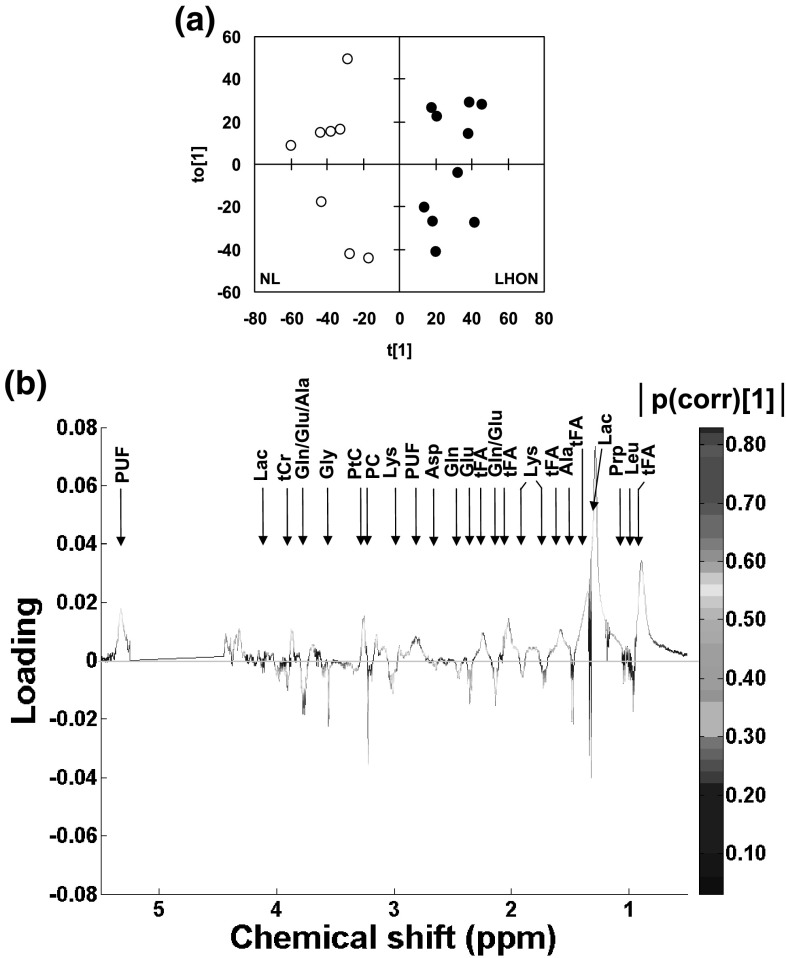
**a, b** OPLS-DA of 1D ^1^H-NMR spectra of LHON vs. NL. NL, n = 8, vs. LHON, n = 10. R2X = 0.60, R2Y = 0.88 and Q2 = 0.58, 1 predictive and 2 orthogonal components. **a** Scores plot (t[1] vs. t[0]). White dots, NL; black dots, LHON. **b** Loadings plot on the predictive component. Main assignment is given. Full assignment and metabolite abbreviations, see Table 1; positive loading, increased in LHON; negative loading, decreased in LHON; color-coding, strength of the correlation coefficient

With OPLS-DA, the use of a single 2-class covariable only provided statistically significant results. First, OPLS-DA of 1D ^1^H NMR spectra (LHON vs. NL, n = 10 vs. n = 8) yielded a good quality model (R2X = 0.60, R2Y = 0.88 and Q2 = 0.58), with one predictive and 2 orthogonal components. The scores plot showed a clear separation between classes (Fig. 1a).

**Table 1:** OPLS-DA of 1D ^1^H-NMR spectra of LHON vs. NL and L-RSV vs. LHON

Chemical shift (ppm)	Metabolite assignment		VIP (pcorr[1])	
	Abbreviation	Full name	LHON vs. NL	L-RSV vs. LHON
5.33	PUF	Polyunsaturated FA (CH = CH)	1.28 (+ 0.51)	1.45 (− 0.40)
4.20	PC	Phosphocholine (CH_2_–O)	–	1.89 (+ 0.55)
4.12	Lac	Lactate (CH)	–	1.76 (+ 0.50)
4.06	MyI	Myoinositol (2-CH)	–	1.26 (+ 0.33)
3.99	PE	Phosphoethanolamine (CH_2_–O)	1.16 (− 0.52)	1.50 (+ 0.41)
3.92	tCr	Total creatine (CH_2_)	1.67 (− 0.80)	1.57 (+ 0.44)
3.77	Glu/Gln/Ala	Glutamate/glutamine/alanine (CH)	1.79 (− 0.86)	2.07 (+ 0.61)
3.62	PC	Phosphocholine (CH_2_–N)	1.34 (− 0.59)	1.72 (+ 0.49)
3.56	Gly	Glycine (CH_2_)	1.61 (− 0.76)	2.08 (+ 0.61)
3.53	MyI	Myoinositol (1,3-CH)	–	2.44 (+ 0.73)
3.43	Tau	Taurine (N–CH_2_)	–	1.29 (− 0.34)
3.26	PtC	Phosphatidylcholine ((CH_3_)_3_–N)	1.36 (+ 0.66)	1.12 (+ 0.29)
3.22	PC	Phosphocholine ((CH_3_)_3_–N)	1.45 (− 0.66)	1.86 (+ 0.54)
3.20	Cho	Choline ((CH_3_)_3_–N)	–	1.30 (− 0.34)
3.03	tCr	Total creatine (CH_3_)	1.60 (− 0.76)	1.07 (+ 0.25)
2.82	PUF	Polyunsaturated FA (=CH–CH^2^–CH=)	1.76 (+ 0.85)	1.91 (− 0.56)
2.64	Asp	Aspartate (β-CH_2_)	1.34 (− 0.64)	1.35 (+ 0.36)
2.55	GSx	Total glutathione (γ-CH_2_)	–	1.23 (− 0.35)
2.44	Gln	Glutamine (γ-CH_2_)	1.44 (− 0.63)	1.32 (+ 0.35)
2.35	Glu	Glutamate (γ-CH_2_)	1.45 (− 0.67)	1.17 (+ 0.35)
2.25	tFA	Total FA (CH_2_–CO)	1.72 (+ 0.81)	1.72 (− 0.49)
2.12	Glu/Gln/GSx	Glutamate/glutamine/glutathione (β-CH_2_)	1.55 (− 0.70)	1.47 (+ 0.40)
2.03	tFA	Total FA (CH_2_–CH=)	1.56 (+ 0.72)	1.29 (− 0.33)
1.90	Lys	Lysine (β–CH_2_)	1.05 (− 0.42)	1.28 (+ 0.33)
1.72		Lysine (δ-CH_2_)	1.15 (− 0.41)	–
1.59	tFA	Total FA (CH_2_–CH_2_–CO)	1.57 (+ 0.72)	1.08 (− 0.24)
1.47	Ala	Alanine (CH_3_)	1.46 (− 0.70)	1.81 (+ 0.52)
1.33	Lac	Lactate (CH_3_)	–	1.76 (+ 0.50)
1.29	tFA	total FA (–(CH_2_)_n_–)	1.58 (+ 0.75)	1.03 (− 0.24)
1.05	Prp	Propionate (CH_3_)	1.10 (− 0.49)	1.80 (+ 0.52)
0.96–1.02	Leu/Ileu/Val	Leucine/isoleucine/valine (CH_3_)	1.04 (− 0.44)	1.58 (+ 0.44)
0.90	tFA	Total FA (CH_3_)	1.59 (+ 0.73)	1.10 (− 0.26)

The first column gives chemical shift of 1D ^1^H-NMR signals. The second and third columns give signal assignment with abbreviation and full name of the metabolite(s). The last 2 columns give VIP when > 1 (statistically significant variation), with p(corr)[1] in parentheses

Dash, not significant. The sign of p(corr)[1] indicates the direction of variation: negative, decrease; positive, increase

In the loading plot (Fig. 1b; Table 1), several NMR signals were increased (VIP > 1 and pcorr[1] > + 0.40) in LHON, including those of tFA, PUF and PtC.

Several NMR signals were decreased in LHON including those of several AAs: Gly, Ala, Glu, Gln, Asp, Lys, and collectively Glu/Gln/Ala/Lys, Glu/Gln and Leu/Ileu/Val. Other signals were decreased including those of tCr, PC, PE and Prp.

**Fig. 2:**
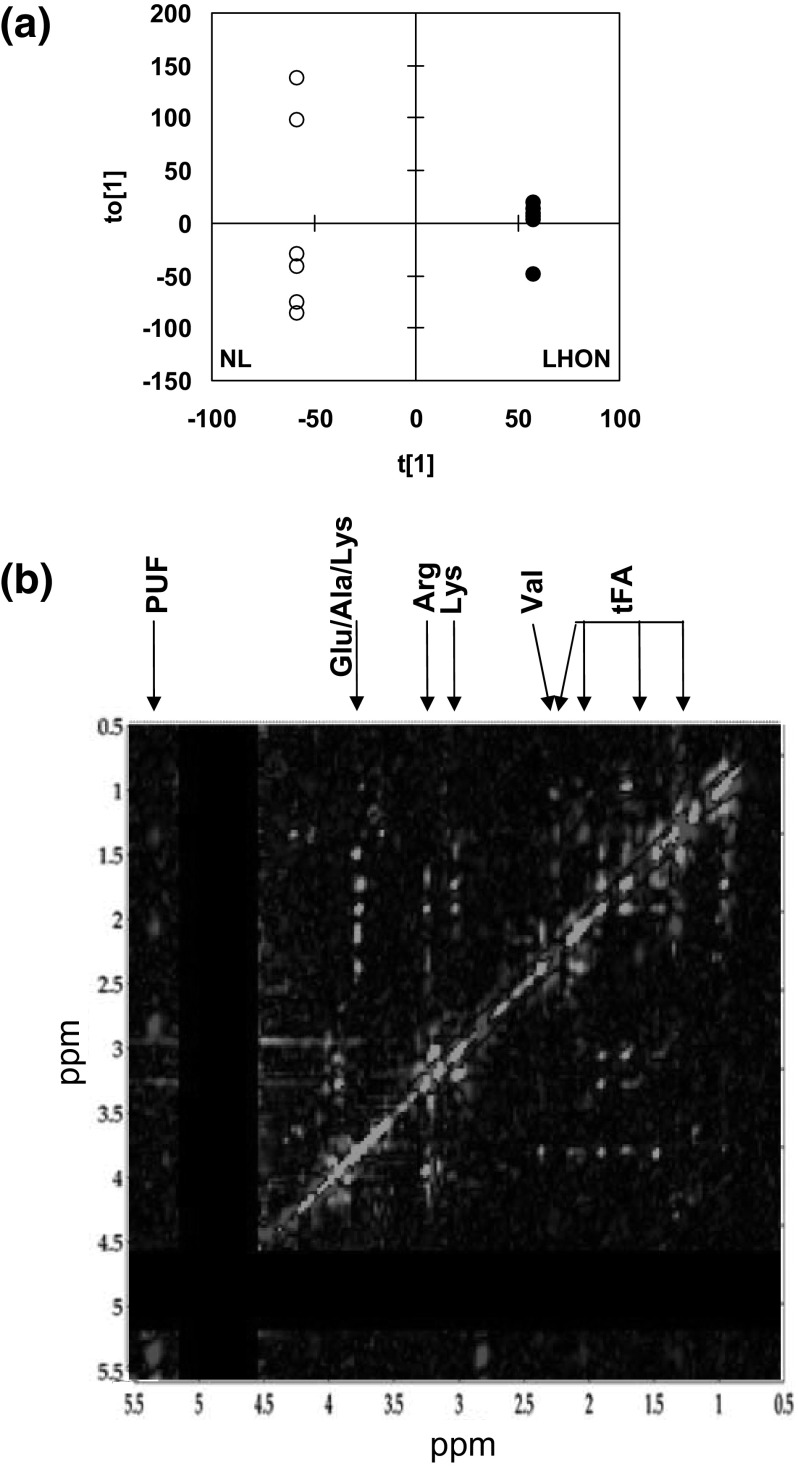
**a, b** OPLS-DA of 2D TOCSY spectra of LHON vs. NL. NL, n = 6, vs. LHON, n = 6. R2X = 0.85, R2Y = 1 and Q2 = 0.42, 1 predictive and 7 orthogonal components. **a** Scores plot (t[1] vs. t[0]). White dots, NL; black dots, LHON. **b** Loadings plot on the predictive component. It is color-coded according to the sign and strength of loadings. Main assignment of loadings is given. Full assignment and metabolite abbreviations, see Table 2; red loading, increased in LHON; green loading, decreased in LHON; biphasic, HEPES which chemical shifts are sensitive to physicochemical conditions

Second, we applied OPLS-DA to 2D TOCSY spectra (LHON vs. NL, n = 6 vs. n = 6). The obtained model had good quality (R2X = 0.85, R2Y = 1 and Q2 = 0.42), with one predictive and 7 orthogonal components. The scores plot showed a clear separation between classes (Fig. 2a). In the loading plot (Fig. 2b; Table 2), some off-diagonal signals were significantly increased in LHON including those of tFA and PUF. Most significantly decreased off-diagonal signals in LHON included those of several amino acids: Val, Arg, Ala, Lys, Asp, Ser and Phe.

**Table 2:** OPLS-DA of 2D TOCSY spectra of LHON vs. NL

Chemical shift (ppm)	Metabolite		VIP (pcorr[1])
	Abbreviation	Full name	LHON vs. NL
5.33 × 2.82	PUF	Polyunsaturated FA	1.49 (+ 0.56)
5.33 × 2.03			1.69 (+ 0.65)
5.33 × 1.29			1.60 (+ 0.61)
4.20 × 3.58	PC	Phosphocholine	1.37 (− 0.49)
4.17 × 2.03	Pro	Proline	1.19 (− 0.43)
4.12 × 1.33	Lac	Lactate	*–*
4.06 × (3.53–3.61)	MyI	Myoinositol	*–*
(3.53–3.61) × 3.27			*–*
3.99 × 3.22	PE	Phosphoethanolamine	1.19 (− 0.40)
3.99 × 3.13	Phe	Phenylalanine	1.39 (− 0.50)
3.99 × (2.88–2.95)	Asn	Asparagine	1.19 (− 0.49)
3.97 × 3.83	Ser	Serine	1.44 (− 0.55)
3.89 × (2.65–2.75)	Asp	Aspartate	1.36 (− 0.50)
3.58 × 1.33	Thr	Threonine	1.15 (− 0.41)
3.77 × 2.35	Glu	Glutamate	1.24 (− 0.48)
2.35 × (2.04–2.11)			1.10 (− 0.40)
3.77 × 1.90	Lys	Lysine	1.39 (− 0.51)
3.77 × 1.71			1.49 (− 0.56)
3.77 × 1.46	Ala	Alanine	1.47 (− 0.56)
3.43 × 3.27	Tau	Taurine	–
3.22 × (1.65–1.90)	Arg	Arginine	1.59 (− 0.61)
2.55 × 2.17	GSx	Total glutathione	1.31 (− 0.47)
2.44 × 2.14	Gln	Glutamine	1.20 (− 0.41)
2.28 × 1.00	Val	Valine	1.63 (− 0.62)
1.71 × 0.95	Leu	Leucine	1.18 (– 0.40)
1.29 × 2.25	tFA	Total FA	1.25 (+ 0.43)
1.29 × 2.03			1.29 (+ 0.48)
1.29 × 0.90			1.33 (+ 0.51)

The first column gives chemical shifts of 2D TOCSY off-diagonal signals. The second and third columns give assignment with abbreviation and full name of the metabolite. The last column gives VIP when > 1 (statistically significant variation), with p(corr)[1] in parentheses

Dash, not significant. The sign of p(corr)[1] indicates the direction of variation: negative, decrease; positive, increase

Then, we applied univariate analysis and OPLS-DA to the set of quantified metabolites. Using univariate statistics (Table 3), lipid derivatives (tFA, PUF and PtC) were increased in LHON (+ 50 to + 117%, LHON vs. NL, all p < 0.05). AA were decreased in LHON, including Phe, Tyr, Gly and Ala (− 24 to − 40%, LHON vs. NL, p < 0.05). Also Prp was decreased (− 41%, LHON vs. NL, p < 0.05). OPLS-DA of quantified metabolites yielded a good quality model (RX2 = 0.59, RY2 = 0.91 and Q2 = 0.75) with 1 predictive and 2 orthogonal components. Significantly increased metabolites in LHON were PUF, PtC, tFA and MyI, and significantly decreased metabolites were Gln, Ala, Gly, Phe, Tyr and Prp (Fig. S2a,c, Table S2).

**Table 3:** Relative concentrations of 1D ^1^H-NMR quantified metabolites

Metabolite		Chemical shift (ppm)	Concentration relative to NL (NL to LHON) or LHON (L-IDE and L-RSV)					
Abbreviation	Full name		NL (n = 8)	N-IDE (n = 7)	N-RSV (n = 6)	LHON (n = 10)	L-IDE (n = 8)	L-RSV (n = 9)
ATP	Adenosine triphosphate	8.54s; 8.12; 6.15	1.00 (1.08)	1.23 (0.80)	1.94 (1.56)	0.94 (0.51)	1.25 (0.40)	1.66 (1.14)
For	Formate	8.46s	1.00 (0.81)	0.90 (1.13)	0.74 (0.39)	1.16 (1.39)	0.56 (0.36)	0.44 (0.26)
AMP	Adenosine monophosphate	8.26s; 8.36; 6.10	1.00 (0.50)	2.66 (3.60)	0.64 (0.35)	3.71 (5.41)	0.12 (0.07)	0.25 (0.27)
Phe	Phenylalanine	7.42m; 7.33	1.00 (0.33)	0.98 (0.28)	1.03 (0.40)	0.76 (0.26)*	1.12 (0.27)	1.32 (0.40)^$^
Tyr	Tyrosine	7.16m; 6.90	1.00 (0.40)	1.00 (0.28)	1.03 (0.44)	0.67 (0.37)*	1.20 (0.27)	1.31 (0.44)
UXP	Uridine phosphate derivatives	5.97m; 7.94	1.00 (0.67)	1.00 (0.39)	1.11 (0.69)	1.14 (0.48)	1.09 (0.29)	1.37 (0.73)
MyI	Myoinositol	4.06m; 3.27; 3.53; 3.61	1.00 (0.63)	2.20 (1.98)	5.41 (3.17)^$$^	2.15 (1.23)	0.88 (0.54)	1.92 (1.03)^$^
tCr	Total creatine	3.92s; 3.03	1.00 (1.28)	0.72 (0.27)	0.92 (0.70)	0.81 (0.28)	1.22 (0.49)	0.85 (0.48)
Gly	Glycine	3.56s	1.00 (0.43)	0.92 (0.25)	0.99 (0.32)	0.69 (0.20)*	1.18 (0.24)	1.20 (0.23)
Tau	Taurine	3.43t ; 3.27	1.00 (0.41)	1.12 (0.44)	1.28 (0.52)	1.47 (0.68)	0.63 (0.17)^#^	0.53 (0.24)^$$^
PtC	Phosphatidylcholine	3.26b; 3.68; 4.35	1.00 (0.36)	1.03 (0.31)	1.04 (0.18)	1.50 (0.37)**	0.77 (0.17)^#^	0.97 (0.30)
PC	Phosphocholine	3.22s; 3.62; 4.18	1.00 (0.76)	0.98 (0.36)	1.47 (0.72)	0.79 (0.46)	1.23 (0.55)	1.42 (0.43)^$^
PUF	Polyunsaturated FA	2.82b; 5.33	1.00 (0.47)	0.93 (0.57)	1.24 (0.62)	2.17 (0.95)**	0.64 (0.32)^#^	0.71 (0.37)
GSx	Total glutathione (reduced + oxidized)	2.55m; 2.12; 2.99; 3.77	1.00 (0.42)	1.03 (0.53)	1.33 (0.56)	1.18 (0.46)	0.79 (0.24)	1.16 (0.57)
Gln	Glutamine	2.44m; 2.14; 3.77	1.00 (0.46)	2.13 (1.74)	1.76 (1.30)	0.68 (0.46)	1.31 (0.88)	1.19 (1.02)
Pyr	Pyruvate	2.37s	1.00 (0.65)	0.95 (0.56)	1.47 (0.67)	0.89 (0.42)	1.03 (0.52)	1.16 (0.78)
Glu	Glutamate	2.35m; 2.04; 2.11; 3.77	1.00 (0.77)	1.05 (0.52)	1.23 (0.76)	0.84 (0.29)	0.97 (0.16)	1.07 (0.58)
Ala	Alanine	1.46d; 3.77	1.00 (0.42)	1.00 (0.28)	1.07 (0.38)	0.60 (0.27)*	1.19 (0.37)	1.16 (0.31)
Lac	Lactate	1.33d; 4.12	1.00 (0.53)	1.30 (0.49)	1.20 (0.29)	1.15 (0.74)	1.03 (0.32)	1.66 (0.50)^$$^
tFA	Total FA (saturated + unsaturated)	1.29b; 1.59; 2.03; 2.25	1.00 (0.32)	0.81 (0.28)	0.91 (0.53)	1.77 (0.83)*	0.64 (0.37)^#^	0.90 (0.42)
Prp	Propionate	1.05t; 2.17	1.00 (0.61)	0.84 (0.33)	1.03 (0.50)	0.59 (0.29)*	1.24 (0.41)	1.16 (0.23)

First and second columns, metabolites; third column, chemical shift of metabolite NMR signals. The signal used for metabolite quantification is given with its coupling pattern specified using a letter: s, singlet; d, doublet; t, triplet; m, multiplet; b, broad signal. Fourth to seventh columns, concentration relative to NL (fold change vs. NL); eighth and ninth columns, concentration relative to LHON (fold change vs. LHON); L-IDE or L-RSV concentration relative to NL = (L-IDE or L-RSV concentration relative to LHON) × (LHON concentration relative to NL)

*p < 0.05 LHON vs. NL; **p < 0.01 LHON vs. NL

^$^p < 0.05 L-RSV vs. LHON; ^$$^p < 0.01 N-RSV vs. NL or L-RSV vs. LHON

^#^p < 0.05, L-IDE vs. LHON (Student’s *t* test)

### IDE reverses lipid changes in LHON fibroblasts

The CI-to-CS activity ratio increased in LHON in response to IDE (87 ± 18 vs. 62 ± 11%, L-IDE vs. LHON, p < 0.05) (Fig. S1).

The OPLS-DA of 1D NMR spectra failed to provide statistically significant models separating N-IDE and NL or L-IDE and LHON.

The quantification of metabolites in response to IDE is given in Table 3. There were no significant changes in N-IDE vs. NL. In contrast, in LHON, IDE decreased tFA, PUF and PtC (L-IDE vs. LHON, n = 8 vs. n = 10, p < 0.05). The OPLS-DA of quantified metabolites yielded no significant model separating N-IDE and NL or L-IDE and LHON.

### RSV partly reverses lipid and amino acid changes in LHON fibroblasts

The CI-to-CS activity ratio increased in LHON in response to RSV (89 ± 25 vs. 62 ± 11%, L-RSV vs. LHON, p < 0.05) (Fig. S1).

The OPLS-DA of 1D NMR spectra did not provide statistically significant models separating N-RSV and NL.

**Fig. 3:**
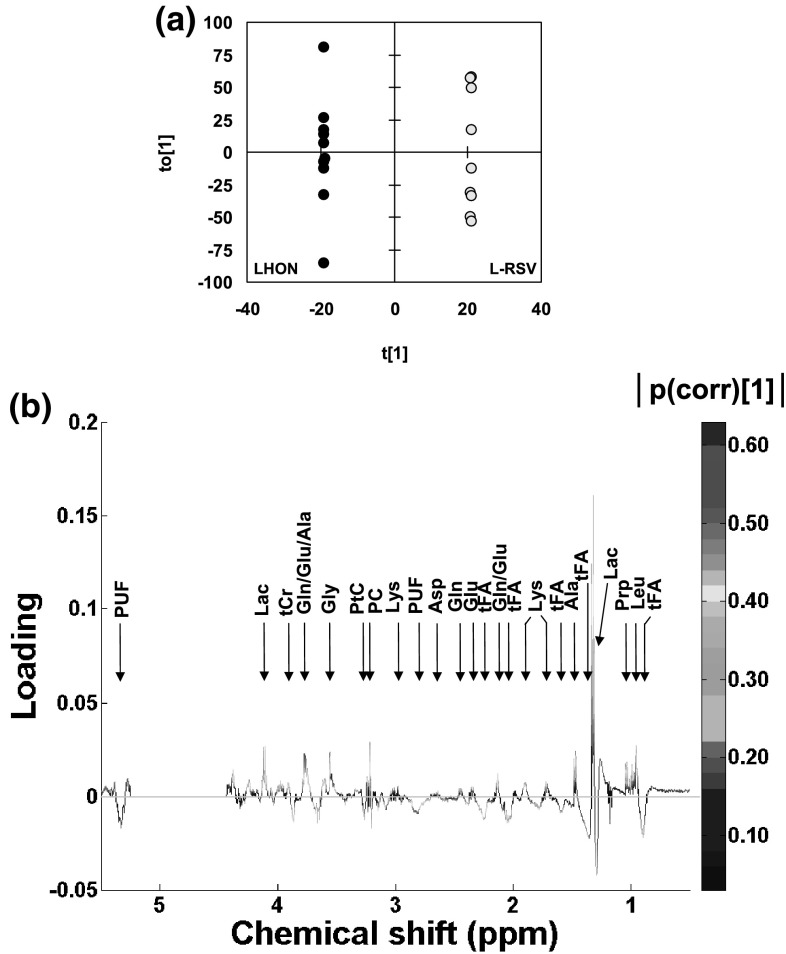
**a, b** OPLS-DA of 1D ^1^H-NMR spectra of L-RSV vs. LHON. LHON, n = 10, vs. L-RSV, n = 9. R2X = 0.96, R2Y = 1 and Q2 = 0.54, 1 predictive and 7 orthogonal components. **a** Scores plot (t[1] vs. t[0]). Black dots, LHON; light grey dots, L-RSV. **b** Loadings plot on the predictive component. Main assignment is given. Full assignment and metabolite abbreviations, see Table 1; positive loading, increased in L-RSV; negative loading, decreased in L-RSV; color-coding, strength of the correlation coefficient

The OPLS-DA of 1D ^1^H-NMR spectra (L-RSV vs. LHON, n = 9 vs. n = 10) yielded a good quality model (R2X = 0.96, R2Y = 1 and Q2 = 0.54), with one predictive and 7 orthogonal components. The scores plot showed a clear separation between classes (Fig. 3a).

In the loading plot (Fig. 3b; Table 1) several NMR signals were decreased under RSV treatment including those of tFA at some spectral positions and PUF. Other NMR signals were increased including those of Glu/Gln, Gly, Ala and branched-chain AA. Three metabolites were specifically increased under RSV: PC, Lac and MyI.

The quantification of metabolites in response to RSV is given in Table 3. In L-RSV vs. LHON, Phe, MyI, PC (all, p < 0.05) and Lac (p < 0.01) were increased and Tau was decreased (p < 0.01). OPLS-DA of quantified metabolites was not significant in N-RSV vs. NL. In L-RSV vs. LHON, OPLS-DA yielded a good quality model (RX2 = 0.48, RY2 = 0.98 and Q2 = 0.77) with one predictive and 2 orthogonal components. Significantly increased metabolites in L-RSV were ATP, MyI, PC, Lac, Ala and Glu, and significantly decreased metabolites were PUF and Tau (Fig. S2b, d, Table S2).

## Discussion

This article demonstrates major metabolic alterations in LHON fibroblasts including the increase of lipids and the decrease of AA. These alterations were corrected by mitochondria-targeted treatments that, at least partly, alleviate CID. Our data provide insights into metabolic and molecular adaptive changes to CID.

### Increased lipid levels in CID

We found increased fatty acid (FA) signals in LHON. Several studies have shown that NMR-visible FA signals originated from FA in triglycerides and cholesterol esters of lipid droplets (LD) (Boren and Brindle 2012). LD are synthesized in the endoplasmic reticulum (ER), and play a major role to store and transport FA, prevent potential cytotoxicity of free FA, and participate to the communication between the ER and mitochondria. Actually, the interaction between mitochondria and ER is very tight, both these organelles being implicated in not only lipid metabolism, but also Ca^2+^ signaling, bioenergetics, inflammation, autophagy and apoptosis.

Alteration in lipid metabolism has been shown in CID. Rotenone, a CI inhibitor, alters FA biosynthesis and beta-oxidation in neuroblastoma cells (Worth et al. 2014) or induces triacylglycerol deposition in muscle-derived cells (He et al. 2013). Also, inhibition of beta-oxidation was reported in CI mutation (Leong et al. 2012).

In LHON, we also found changes in phospholipid derivatives and Prp. PtC was increased and PC decreased, suggesting increased PtC biosynthesis, a condition for LD formation. Prp in the form of propionyl-CoA, is synthesized by incomplete beta-oxidation of odd-chain FA in peroxisomes. Since beta-oxidation in peroxisomes requires reoxidation of NADH by mitochondria, the decrease of Prp could result from the inhibition of beta-oxidation in peroxisomes. Alternatively, Prp is a by-product of the degradation of branched-chain AA that, as discussed below, were decreased in LHON.

Overall, the increase of intracellular levels of lipids, especially FA, in LHON can be related to the reduction of mitochondrial/peroxisomal lipid beta-oxidation and the increase of LD formation.

### Decreased amino acid levels in CID

We found decreased intracellular levels of free AA in LHON, in agreement with several reports in cells with deficient ETC. A NMR metabolomics analysis of an inherited neurodegeneration reported the decrease of Gly and Glu-related AA (Graham et al. 2016). A MS-metabolomics investigation of LHON fibroblasts showed the decrease of proteinogenic AA (Chao de la Barca et al. 2016). A study on pharmacological inhibition of ETC complexes reported the decrease of several AA (Baykal et al. 2008). A MS study of the response of transformed cells to CI inhibitors reported the decrease of Glu and Gln (Janzer et al. 2014).

Intracellular levels of free AA result from the balance between uptake, protein synthesis, oxidation and recycling by autophagy. AA oxidation through the TCA cycle should not be activated in LHON. As well, increased protein synthesis due to mitogenesis should not explain the magnitude of AA variations.

Membrane AA transport often depends on the redox status of the cell and/or requires the availability of ATP. A reduction in the intracellular transport of Glu, through decreased activity of EAAT1, was reported in LHON cybrids (Beretta et al. 2004). In addition, the reduction in plasma membrane import of AA may be caused by AMPK activation, an adaptive mechanism in CID (Distelmaier et al. 2015).

Autophagy is another mechanism that regulates AA levels. Low levels of AA activate autophagy through the inhibition of the mTOR pathway. The accumulation of autophagosomes was reported in LHON, in favor of the activation of autophagy (Dombi et al. 2016). In addition, mitogenesis, another adaptive mechanism in CID, often combines with mitophagy in the control of mitochondrial quality. Completion of the autophagic process requires fusion of autophagosomes with lysosomes, a mechanism that, despite activation of autophagy, may be impaired in neurodegeneration.

Overall, the decrease of intracellular levels of AA in LHON fibroblasts can be related to decreased membrane uptake/cytosolic transport of AA, or activated autophagy.

### Reversion of metabolic changes with mitochondria-targeted treatments

In LHON, IDE increased the activity of CI, in agreement with other reports (Angebault et al. 2011). IDE completely reversed FA changes. IDE is an analogue of ubiquinone and favors bypassing of CI directly to Complex III of the ETC. It is a potent antioxidant and prevents ROS-induced mitochondrial dysfunction. From the above discussion on FA in LHON, the response to IDE probably testifies the improvement of mitochondrial betaoxidation and the reduction of LD formation. In addition, IDE induced no significant effects on AA levels, thus should not target AA transport or mitophagy.

The other treatment, RSV, increased CI activity in LHON, in agreement with other reports in CID (Lopes Costa et al. 2014). FA signals were partially decreased in LHON treated by RSV, in favor of an improvement in mitochondrial/peroxisomal beta-oxidation and/or reduction of LD. This is agreement with reports on RSV (Massimi et al. 2012). RSV increased AA levels. From our discussion about AA levels in LHON, this supports that RSV improves cytosolic AA transport and mitophagy. Indeed several reports showed that RSV increases Glu and AA transporter activity in astrocytes and glioma cells (Bellaver et al. 2016), and activates autophagy (Wu et al. 2011).

Intriguingly, RSV increased markedly Lac level in LHON. This could be due to downregulation of monocarboxylate transporters. However, the beneficial impact of RSV on cellular bioenergetics is quite complex, resulting from changes in redox homeostasis, activation of AMPK, stimulation of mitogenesis and mitophagy, but also from mitochondrial uncoupling and inhibition of ATP synthase/Complex V (de Oliveira et al. 2016). A hypothesis is that RSV could make Complex V to function in reverse as an ATPase (Distelmaier et al. 2015), thus consume ATP of glycolytic origin, increase Lac production and intracellular Lac level. ATPases are also found in lysosome membranes. They may be activated by RSV which increases lysosome fusion with autophagosomes, thus contributing to increase cytosolic AA levels.

### Implications for LHON

The main target of LHON is RGC. Although extrapolation from skin fibroblasts to RGC needs to be cautious, findings of this study support hypotheses on RGC sensitivity to cell death in LHON. Bioenergetics alteration does not satisfactorily explain loss of RGC (Kirches 2011), in agreement with preservation of ATP levels in LHON in our study. Alternatively, reduction of cytosolic AA levels that may originate from decreased plasma membrane uptake could contribute to increase extracellular Glu levels that cause excitotoxicity. Also the increase of LD, autophagosomes, and mitochondria-ER interactions could impair mitochondrial trafficking to the sites of energetic needs, another cause of RGC loss. Importantly, metabolic response to mitochondria-targeted treatments was consistent with (IDE) or in favor of (RSV) the beneficial effect of these agents in LHON.

## Overall conclusions

The present NMR spectroscopy-based metabolomics study shows that LHON fibroblasts exhibit metabolic alterations including intracellular lipid increase and AA decrease. These abnormalities were corrected partly by mitochondria-targeted treatments, and could be explained by adaptive changes to CID, including inhibition of betaoxidation, formation of LD, decreased cytosolic AA transport and activation of autophagy. CID-associated pathways in LHON fibroblasts can help to explain CID expression in LHON, in response to CI-targeted drugs or in diseases including neurodegeneration and cancer.

## Electronic supplementary material

Below is the link to the electronic supplementary material.

Supplementary material 1 (PDF 219 KB)

## Abbreviations

CI: Mitochondrial Complex I
CID: Mitochondrial Complex I deficiency
LHON: Leber’s hereditary optic neuropathy
ETC: Electron transport chain
EAAT1: Excitatory amino acid transporter-1
IDE: Idebenone
RSV: Resveratrol
HRMAS: High resolution magic angle spinning
OPLS-DA: Orthogonal partial least squares-discriminant analysis
VIP: Variable importance in the prediction
FA: Fatty acid
AA: Amino acid
ER: Endoplasmic reticulum
LD: Lipid droplet

## References

[CR1] Angebault C, Gueguen N, Desquiret-Dumas V, Chevrollier A, Guillet V, Verny C (2011). Idebenone increases mitochondrial complex I activity in fibroblasts from LHON patients while producing contradictory effects on respiration. BMC Research Notes.

[CR2] Bayet-Robert M, Loiseau D, Rio P, Demidem A, Barthomeuf C, Stepien G, Morvan D (2010). Quantitative two-dimensional HRMAS 1H-NMR spectroscopy-based metabolite profiling of human cancer cell lines and response to chemotherapy. Magnetic Resonance in Medicine.

[CR3] Baykal AT, Jain MR, Li H (2008). Aberrant regulation of choline metabolism by mitochondrial electron transport system inhibition in neuroblastoma cells. Metabolomics.

[CR4] Bellaver B, Bobermin LD, Souza DG, Rodrigues MDN, de Assis AM, Wajner M (2016). Signaling mechanisms underlying the glioprotective effects of resveratrol against mitochondrial dysfunction. Biochimica et Biophysica Acta (BBA) - Molecular Basis of Disease.

[CR5] Beretta S, Mattavelli L, Sala G, Tremolizzo L, Schapira AHV, Martinuzzi A (2004). Leber hereditary optic neuropathy mtDNA mutations disrupt glutamate transport in cybrid cell lines. Brain: A Journal of Neurology.

[CR6] Boren J, Brindle KM (2012). Apoptosis-induced mitochondrial dysfunction causes cytoplasmic lipid droplet formation. Cell Death and Differentiation.

[CR7] Chao de la Barca JM, Simard G, Amati-Bonneau P, Safiedeen Z, Prunier-Mirebeau D, Chupin S (2016). The metabolomic signature of Leber’s hereditary optic neuropathy reveals endoplasmic reticulum stress. Brain.

[CR8] de Oliveira MR, Nabavi SF, Manayi A, Daglia M, Hajheydari Z, Nabavi SM (2016). Resveratrol and the mitochondria: From triggering the intrinsic apoptotic pathway to inducing mitochondrial biogenesis, a mechanistic view. Biochimica et Biophysica Acta.

[CR9] Distelmaier F, Valsecchi F, Liemburg-Apers DC, Lebiedzinska M, Rodenburg RJ, Heil S (2015). Mitochondrial dysfunction in primary human fibroblasts triggers an adaptive cell survival program that requires AMPK-α. Biochimica et Biophysica Acta (BBA) - Molecular Basis of Disease.

[CR10] Dombi E, Diot A, Morten K, Carver J, Lodge T, Fratter C (2016). The m.13051G > A mitochondrial DNA mutation results in variable neurology and activated mitophagy. Neurology.

[CR11] Graham SF, Kumar PK, Bjorndahl T, Han B, Yilmaz A, Sherman E (2016). Metabolic signatures of Huntington’s disease (HD): (1)H NMR analysis of the polar metabolome in post-mortem human brain. Biochimica et Biophysica Acta.

[CR12] He Q, Wang M, Petucci C, Gardell SJ, Han X (2013). Rotenone induces reductive stress and triacylglycerol deposition in C_2_C_12_ cells. The International Journal of Biochemistry & Cell Biology.

[CR13] Janzer A, German NJ, Gonzalez-Herrera KN, Asara JM, Haigis MC, Struhl K (2014). Metformin and phenformin deplete tricarboxylic acid cycle and glycolytic intermediates during cell transformation and NTPs in cancer stem cells. Proceedings of the National Academy of Sciences of the United States of America.

[CR14] Kirches E (2011). LHON: Mitochondrial mutations and more. Current Genomics.

[CR15] Leong DW, Komen JC, Hewitt CA, Arnaud E, McKenzie M, Phipson B (2012). Proteomic and metabolomic analyses of mitochondrial complex I-deficient mouse model generated by spontaneous B2 short interspersed nuclear element (SINE) insertion into NADH dehydrogenase (ubiquinone) Fe-S protein 4 (Ndufs4) gene. The Journal of Biological Chemistry.

[CR16] Loiseau D, Chevrollier A, Verny C, Guillet V, Gueguen N, Pou de Crescenzo M-A (2007). Mitochondrial coupling defect in Charcot-Marie-Tooth type 2A disease. Annals of Neurology.

[CR17] Lopes Costa A, Le Bachelier C, Mathieu L, Rotig A, Boneh A, De Lonlay P (2014). Beneficial effects of resveratrol on respiratory chain defects in patients’ fibroblasts involve estrogen receptor and estrogen-related receptor alpha signaling. Human Molecular Genetics.

[CR18] Massimi M, Tomassini A, Sciubba F, Sobolev AP, Devirgiliis LC, Miccheli A (2012). Effects of resveratrol on HepG2 cells as revealed by 1H-NMR based metabolic profiling. Biochimica et Biophysica Acta (BBA) - General Subjects.

[CR19] Mathieu L, Costa AL, Le Bachelier C, Slama A, Lebre A-S, Taylor RW (2016). Resveratrol attenuates oxidative stress in mitochondrial Complex I deficiency: Involvement of SIRT3. Free Radical Biology & Medicine.

[CR20] Mimaki M, Wang X, McKenzie M, Thorburn DR, Ryan MT (2012). Understanding mitochondrial complex I assembly in health and disease. Biochimica et Biophysica Acta.

[CR21] Morgan PG, Higdon R, Kolker N, Bauman AT, Ilkayeva O, Newgard CB (2015). Comparison of proteomic and metabolomic profiles of mutants of the mitochondrial respiratory chain in *Caenorhabditis elegans*. Mitochondrion.

[CR22] Worth AJ, Basu SS, Snyder NW, Mesaros C, Blair IA (2014). Inhibition of neuronal cell mitochondrial complex I with rotenone increases lipid β-oxidation, supporting acetyl-coenzyme A levels. The Journal of Biological Chemistry.

[CR23] Wu Y, Li X, Zhu JX, Xie W, Le W, Fan Z (2011). Resveratrol-activated AMPK/SIRT1/autophagy in cellular models of Parkinson’s disease. Neuro-Signals.

[CR24] Xu Q, Vu H, Liu L, Wang T-C, Schaefer WH (2011). Metabolic profiles show specific mitochondrial toxicities in vitro in myotube cells. Journal of Biomolecular NMR.

[CR25] Zhang L-N, Hao L, Wang H-Y, Su H-N, Sun Y-J, Yang X-Y (2015). Neuroprotective effect of resveratrol against glutamate-induced excitotoxicity. Advances in Clinical and Experimental Medicine: Official Organ Wroclaw Medical University.

