# Supplementary material for: NMR metabolomics of fibroblasts with inherited mitochondrial Complex I mutation reveals treatment-reversible lipid and amino acid metabolism alterations
Source: Metabolomics. 2018 Mar 22;14(5):55. doi: 10.1007/s11306-018-1345-9 (PMC5968059; doi:10.1007/s11306-018-1345-9)
Supplement: Supplementary file 1 — Supplementary material 1 (PDF 219 KB) [file 11306_2018_1345_MOESM1_ESM.pdf]

# Supplementary material

## Supplementary figure legends

**Fig. S1** CI-to-CS activity ratio

Ratio of the activity of CI to the activity of citrate synthase (CS) in NL and LHON fibroblasts exposed to solvent (NL, LHON), 10 μM idebenone (N-IDE, L-IDE) and 50 μM resveratrol (N-RSV, L-RSV).

*, $p<0.01$, LHON vs. NL, n=5 vs n=4; $, $p<0.05$, L-IDE vs. LHON, n=5 vs n=5; #, $p<0.05$, L-RSV vs. LHON, n=5 vs n=5 (Student's t-test).

**Fig. S2a-d** OPLS-DA of quantified metabolites of LHON vs. NL and L-RSV vs. LHON

a LHON vs. NL. Scores plot (t[1] vs. t[0]). Black dots, LHON; white dots, NL.

b L-RSV vs. LHON. Scores plot (t[1] vs. t[0]). Black dots, LHON; light grey dots, L-RSV.

c LHON vs. NL. Loadings plot on the predictive component. Metabolite abbreviation, see Table 3; positive loading, increased in LHON; negative loading, decreased in LHON; error bars, cross-validated SE; *, VIP>1.

d L-RSV vs. LHON. Loadings plot on the predictive component. Metabolite abbreviation, see Table 3; positive loading, increased in L-RSV; negative loading, decreased in L-RSV; error bars, cross-validated SE; *, VIP>1.

**Table S1** Demographic and genetic data of patients

| Patient | Age (years) | Sex | Mutation | Heteroplasmy(%) | Haplogroup |
|---|---|---|---|---|---|
| 1 | 20 | M | m.11778G>A | 90 | K |
| 2 | 32 | M | m.11778G>A | 100 | V |
| 3 | 44 | M | m.11778G>A | 81 | J |
| 4 | 47 | M | m.11778G>A | 100 | H |
| 5 | 52 | M | m.11778G>A | 100 | H |

**Table S2** OPLS-DA of quantified metabolites of LHON vs. NL and L-RSV vs. LHON

| **Metabolite (n=21)** | **VIP (pcorr[1])** | |
|---|---|---|
| | **LHON vs. NL** | **L-RSV vs. LHON** |
| ATP | - | 1.26(+0.53) |
| For | - | - |
| AMP | - | - |
| Phe | 1.03(-0.34) | - |
| Tyr | 1.03(-0.37) | - |
| UXP | - | - |
| MyI | 1.13(+0.59) | 1.31(+0.53) |
| Gly | 1.13(-0.47) | - |
| tCr | - | - |
| Tau | - | 1.46(-0.64) |
| PC | - | 1.50(+0.64) |
| PtC | 1.20 (+0.56) | - |
| PUF | 1.50(+0.78) | 1.13(-0.51) |
| GSx | - | - |
| Gln | 1.29 (-0.66) | - |
| Pyr | - | - |
| Glu | - | 1.02(+0.17) |
| Ala | 1.21(-0.50) | 1.00(+0.26) |
| Lac | - | 1.01(+0.45) |
| tFA | 1.33(+0.69) | - |
| Prp | 1.01 (-0.35) | - |

The first column gives the metabolite abbreviation (full name, see Table 3). The second and third columns give VIP when >1 (statistically significant variation), with p(corr)[1] in parentheses. Dash, not significant. The sign of p(corr)[1] indicates the direction of variation: negative, decrease; positive, increase.

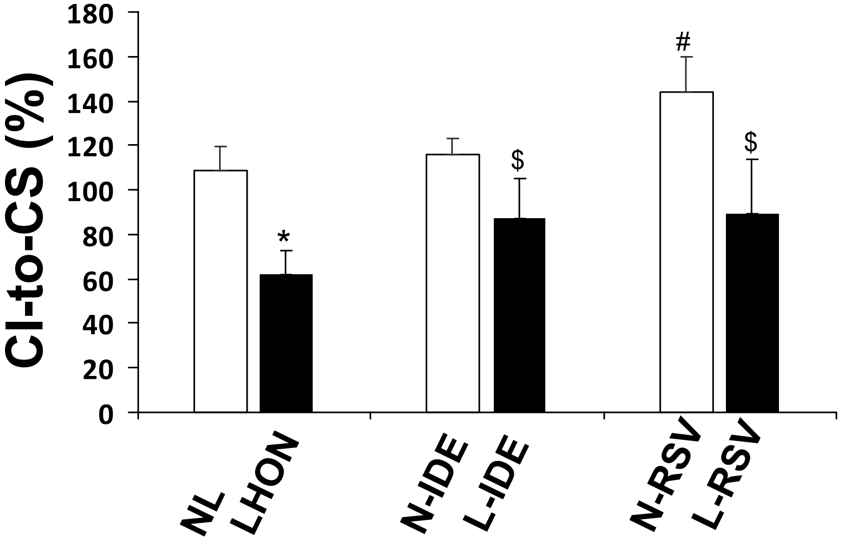
CI-to-CS (%)
180
160
140
120
100
80
60
40
20
0
*
$
#
$
NL
LHON
N-IDE
L-IDE
N-RSV
L-RSV

Fig.S1

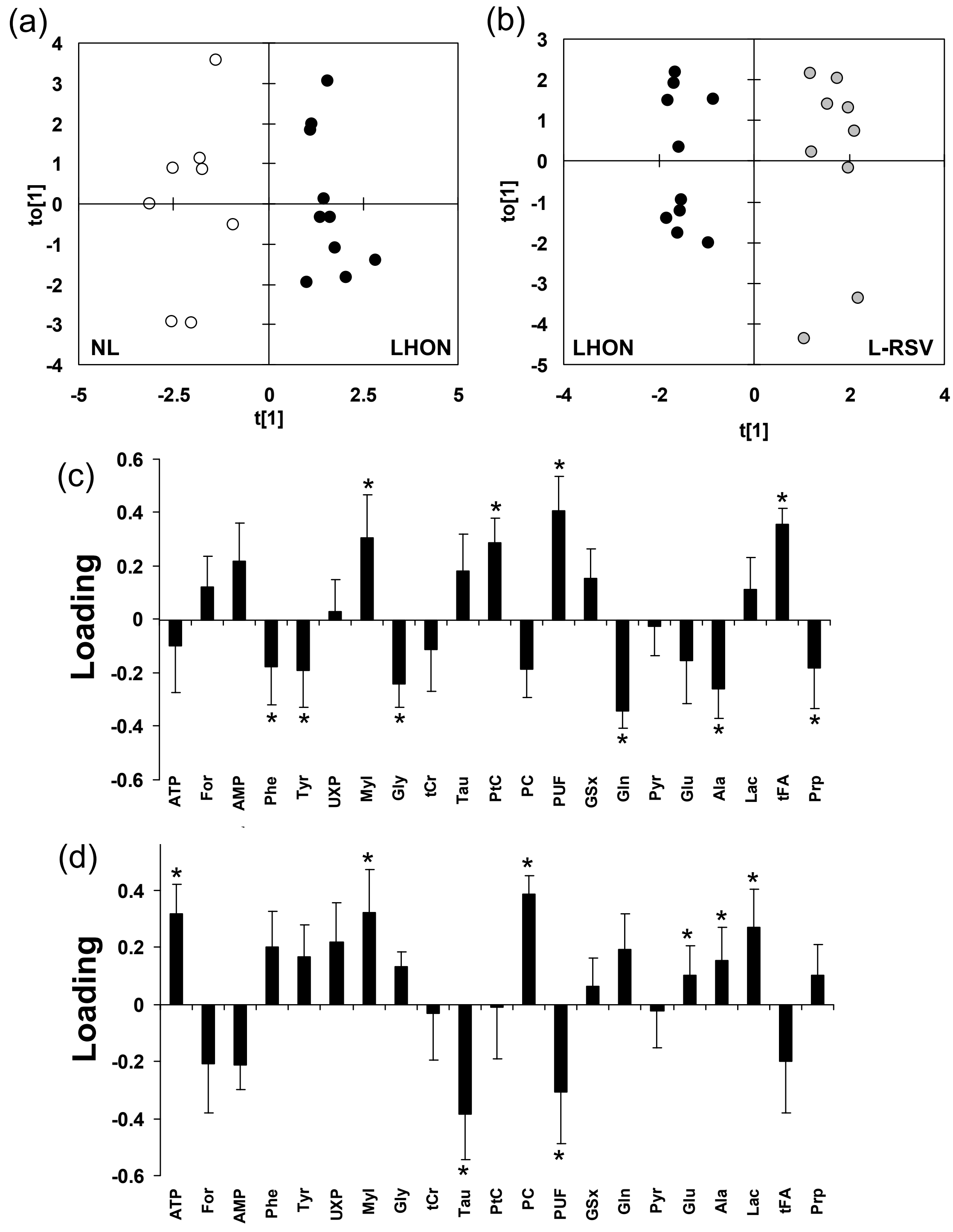
(a)
NL
LHON
to[1]
t[1]
(b)
LHON
L-RSV
to[1]
t[1]
(c)
Loading
ATP
For
AMP
Phe
Tyr
UXP
Myl
Gly
tCr
Tau
PtC
PC
PUF
GSx
Gln
Pyr
Glu
Ala
Lac
tFA
Prp
(d)
Loading

**Fig.S2**
